# The Application of Human-Centered Design Approaches in Health Research and Innovation: A Narrative Review of Current Practices

**DOI:** 10.2196/28102

**Published:** 2021-12-06

**Authors:** Irene Göttgens, Sabine Oertelt-Prigione

**Affiliations:** 1 Department of Primary and Community Care Radboud University Medical Center Nijmegen Netherlands

**Keywords:** human-centered design, design thinking, user-centered design, design-based research, methodology, review, mobile phone

## Abstract

**Background:**

Human-centered design (HCD) approaches to health care strive to support the development of innovative, effective, and person-centered solutions for health care. Although their use is increasing, there is no integral overview describing the details of HCD methods in health innovations.

**Objective:**

This review aims to explore the current practices of HCD approaches for the development of health innovations, with the aim of providing an overview of the applied methods for participatory and HCD processes and highlighting their shortcomings for further research.

**Methods:**

A narrative review of health research was conducted based on systematic electronic searches in the PubMed, CINAHL, Embase, Cochrane Library, Web of Science, PsycINFO, and Sociological Abstracts (2000-2020) databases using keywords related to *human-centered design*, *design thinking* (DT), and *user-centered design* (UCD). Abstracts and full-text articles were screened by 2 reviewers independently based on predefined inclusion criteria. Data extraction focused on the methodology used throughout the research process, the choice of methods in different phases of the innovation cycle, and the level of engagement of end users.

**Results:**

This review summarizes the application of HCD practices across various areas of health innovation. All approaches prioritized the user’s needs and the participatory and iterative nature of the design process. The design processes comprised several design cycles during which multiple qualitative and quantitative methods were used in combination with specific design methods. HCD- and DT-based research primarily targeted understanding the research context and defining the problem, whereas UCD-based work focused mainly on the direct generation of solutions. Although UCD approaches involved end users primarily as testers and informants, HCD and DT approaches involved end users most often as design partners.

**Conclusions:**

We have provided an overview of the currently applied methodologies and HCD guidelines to assist health care professionals and design researchers in their methodological choices. HCD-based techniques are challenging to evaluate using traditional biomedical research methods. Previously proposed reporting guidelines are a step forward but would require a level of detail that is incompatible with the current publishing landscape. Hence, further development is needed in this area. Special focus should be placed on the congruence between the chosen methods, design strategy, and achievable outcomes. Furthermore, power dimensions, agency, and intersectionality need to be considered in co-design sessions with multiple stakeholders, especially when including vulnerable groups.

## Introduction

### Background

Health systems are experiencing a progressive imbalance between available resources and increasing needs. The world population is growing, and the incidence of chronic diseases is rising; however, the funds allocated to health care are limited [1,2]. The need to provide optimized, individualized, and person-centered care is growing. Addressing these competing needs and complex problems requires novel and creative approaches for the development of health care solutions. Design approaches to health care promise to aid the development of innovative, effective, and person-centered solutions to health challenges, supporting the realization of a future for health care that is preventative, personalized, and participatory in nature [3,4]. Different medical disciplines are increasingly applying human-centered design (HCD) to a range of complex questions, from process optimization to product design and social innovation [5-7]. HCD is often described as an iterative, collaborative, and people-centered approach for designing products, services, and systems and is argued to be particularly well-suited for solving complex challenges [8]. In recent years, a growing number of health care professionals have applied HCD to develop person-centered health care solutions in collaboration with patients [9]. For example, the Department of Obstetrics and Gynecology at Mayo Clinic used HCD to develop a new prenatal care model designed to demedicalize a healthy pregnancy experience [10]. By enabling women to meaningfully participate in the process through the use of self-measurement tools, their levels of engagement, sense of control, confidence, and reassurance significantly increased. Another example is the nurse-led quality improvement project at Kaiser Permanente Northern California. HCD principles were used for a patient-centered approach to improve inpatient pain management. The experiences of frontline nurses, patients, and managers were collected, evaluated, and applied to improve the care experience of patients and the work experience of care providers [11].

However, the application of HCD beyond the design sector and its adoption in health research is still in its infancy [4,12]. The number of HCD studies that describe a full project cycle is limited, and even fewer publications focus on the evaluation of research projects that use HCD [13]. A recent scoping review on the application of HCD in global health provided a first overview of its application and health outcomes in public health. The review concluded that increased methodological rigor in the application and reporting of HCD is needed to allow for more acceptance and integration of design practices into research and development [13,14]. However, currently, there is no integral collection of HCD approaches and methods used in the development of health innovations. We performed this review to fill this gap.

HCD evolved from the collaborative design movement and covers a range of overlapping collaborative processes and techniques such as, and not limited to, participatory design, ethnography, cocreation, contextual design, co-design, and empathic design. These processes share several principles: the active involvement of users, an iterative design process, and the organization of multidisciplinary teamwork [15-17]. The term HCD, as a collaborative multimethod approach, is often used interchangeably with terms such as *design thinking* (DT) or *user-centered design* (UCD) because of their similar design philosophies. DT is an approach that prioritizes developing empathy for users, working in collaborative multidisciplinary teams, and using an iterative process with *rapid prototyping* techniques for potential solutions [18]. Similarly, UCD, although deeply rooted in human-computer interactions, is described as both a philosophy and a set of methods in which end users actively influence and are involved in the design process [13]. As these principles are akin to those of HCD, this review includes both DT and UCD as variations that apply HCD principles to further explore their similarities and differences.

### Objective

In this review, we systematically explore the following question: how is HCD, and the closely related approaches of DT and UCD, applied in the development of innovations for health research? We specifically focus on the applied research methodologies and design methods used throughout the study. We investigate the level of engagement of end users during the HCD design processes. As a result, we provide an overview of the current application practices of HCD in health research and a practice-oriented collection of the used design methods to aid future researchers in their choice of methodology.

## Methods

### Overview

A total of 2 librarians, 1 from medical sciences and 1 from social sciences, assisted with the development of a search strategy and the selection of the appropriate databases. Our research included health research related to biomedical, nursing, and allied health and public health sciences. We performed multiple test runs to optimize the search strategy before the first search in July 2019. A final search was performed in August 2020 to update the included publications. The protocol for this review can be found in Multimedia Appendix 1.

### Search Strategy

We performed electronic searches in the following databases: PubMed, CINAHL, Embase, Cochrane Library, Web of Science, PsycINFO, and Sociological Abstracts. Gray literature searches were not included. We searched for studies in the English language that were published between 2000 and 2020. For medical databases, the following terms were used: *Human-centered* OR *Human-centred* OR *User-centered* OR *User-centred* AND *Design OR approach* OR *Design thinking.* For nonmedical databases, the following search terms were added: *Health* OR *Medic* OR *Clinic*. The exact search algorithms per database can be found in Multimedia Appendix 2.

### Eligibility Criteria

We included health research studies that applied HCD, UCD, or DT; focused on the development process of a health innovation; and provided a detailed description of the design process, which included the applied process steps or phases, the applied design methods per process step or phase, and a description of the involved design team and end users. We excluded studies if they did not focus on the design process and did not provide a detailed description of the design processes and the HCD, DT, or UCD methods used in the study. No specific criteria were formulated related to the end user population.

We conceptualized a *health innovation* as it is applied within the context of health research according to the World Health Organization concept of “Health innovation identifies new or improved health policies, systems, products and technologies, and services and delivery methods that improve people’s health and wellbeing.”

### Screening and Data Extraction

We downloaded relevant papers on the Endnote bibliographic software (Clarivate Analytics) and removed duplicates. We then uploaded the Endnote database with the remaining papers on Rayyan, a web application that supports the initial screening of publication titles and abstracts [19]. A total of 2 reviewers independently screened the titles and abstracts for inclusion eligibility and subsequently screened the full-text articles independently for inclusion. We resolved disagreements through discussions. To determine the level of agreement, both Cohen κ value and the percentage of agreement were calculated.

### Data Retrieval and Analysis

We conducted a stepwise analysis of the included publications, focusing on (1) *study characteristics*, including *design phases and methods*, (2) *level of end user involvement*, and (3) *quality assessment.*

#### Study Characteristics

We extracted the following data from each article: year of publication, first author, title of the study, aim of the study, end user of the innovation, type of innovation, study design, design approach, design approach reference, design process phases, applied research and design methods, and the design-based problem-solving strategy.

For the classification of the applied *qualitative and quantitative research and design methods*, research methods were defined as “methods traditionally used within scientific research, oriented towards understanding” and design methods were defined as “methods not traditionally used with scientific research, oriented towards action or solution creation for defined problems” [20,21]. These distinctions were made based on the discussions between the authors. To define the design-based problem-solving strategy, we used the categories of problem-focused strategy (PFS) versus solution-focused strategy (SFS). Studies that use a PFS aim to define or reframe the problem before formulating possible solutions. Studies that use an SFS approach focus on the development of a predefined solution, investing little time in defining or reframing the problem [22].

#### Level of Involvement of the End User

To define the level of engagement of the end user, we adopted a modified framework proposed by Druin [23], which was originally used to categorize the participating role of children in a design process. The participating roles were *users, testers, informants,* or *design partners*, with increased levels of involvement for each role. *Users* help researchers and designers understand the problem context and user needs. The role of *testers* builds upon this role by including end users as part of the initial or functional prototype testing. In the role of *informants*, end users are involved during various stages of the design process, and they contribute to idea generation and provide feedback on the initial and functional prototypes. In the role of *design partners,* end users are considered equal partners of the design team and are involved at all stages of the design process and fully included during the decision-making processes.

#### Quality Assessment

We assessed the quality of reporting and analysis of the study designs using the Mixed Methods Appraisal Tool (MMAT), which allows for the appraisal of studies for literature reviews that include qualitative, quantitative, and mixed methods studies [24]. As most HCD studies apply a multimethod approach, we considered this tool fit for purpose. The MMAT contains 2 general screening questions and 5 study design–specific criteria for assessing quantitative and qualitative studies. For mixed methods studies, we applied both sets of criteria, in addition to 5 specific mixed methods criteria. The scores per item could vary between *yes* (criterion is met), *no* (criterion is not met), and *can’t tell* (paper did not report appropriate information to rate this criterion).

One of the authors first performed the data retrieval and conducted the stepwise analysis described above. Subsequently, both authors reviewed and discussed the results.

## Results

### Overview

In the following sections, we have provided an overview of the literature search results and the study characteristics of the included studies. Subsequently, several aspects of the studies have been highlighted, including the applied design theories, guidelines, strategies, and design process steps. Furthermore, we evaluated the applied research and design methods and the role in which end users were involved throughout the studies.

**Figure 1:**
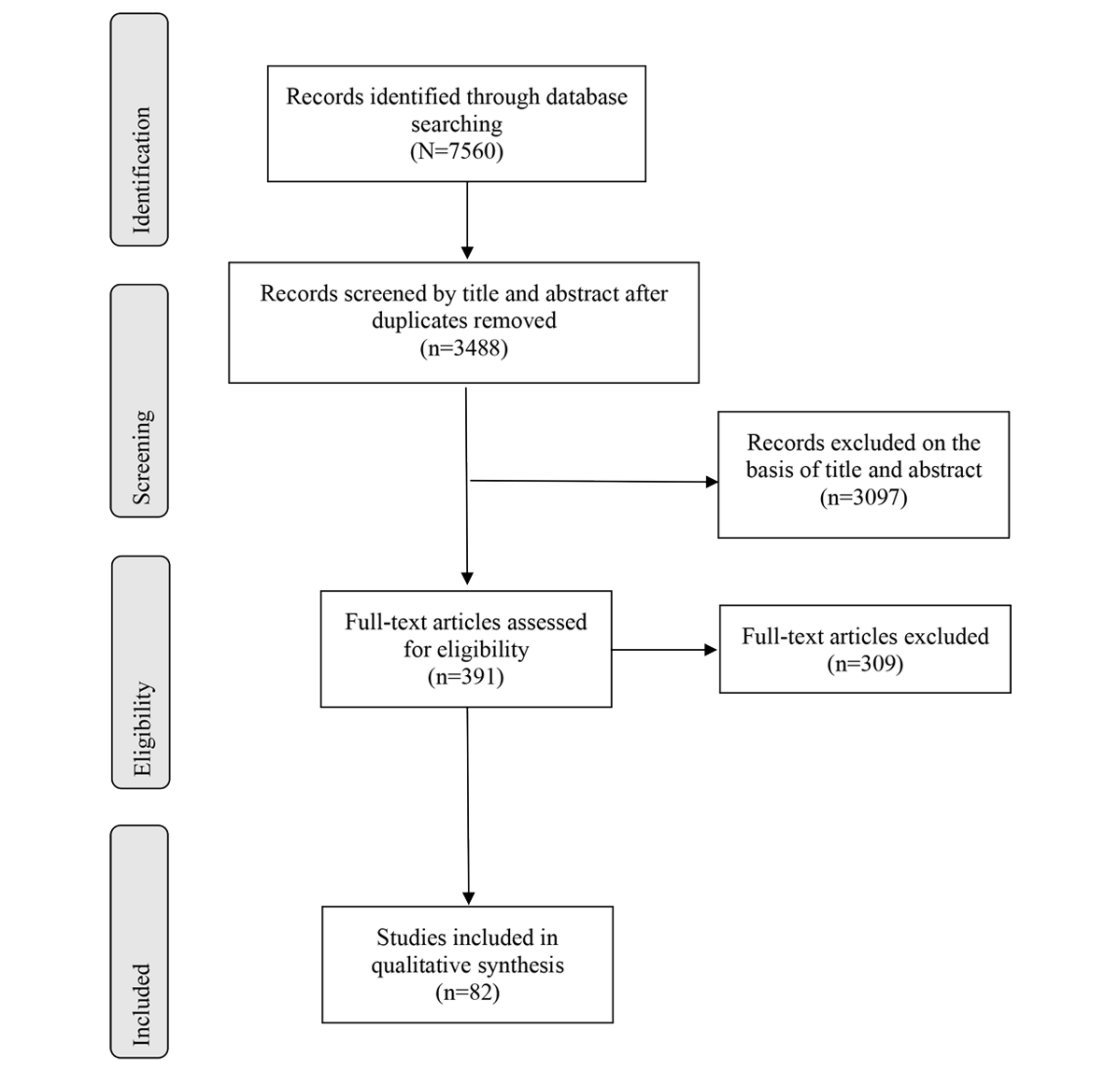
PRISMA (Preferred Reporting Items for Systematic Reviews and Meta-Analyses) flowchart of the screening process.

Our literature search identified 7560 records. Of the 7560 papers, after the removal of 4072 (53.86%) duplicates and exclusions on the basis of abstract for 3097 (40.97%) papers and full text for 309 (4.09%) papers, 82 (1.08%) articles were included in the final analysis (Figure 1). Interrater agreement on the inclusion and exclusion of the studies was 96%, with Cohen κ=0.81.

### Study Characteristics

Of the 82 included papers, 68 (83%) were published between 2015 and 2020. Most studies originated from the United States (34/82, 41%), the Netherlands (7/82, 9%), the United Kingdom (6/82, 7%), and Canada (6/82, 7%). Most studies focused on patients as the end users and developed health innovations with a focus on improving patient care. The most common type of study design was a mixed methods strategy (47/82, 57%), that is, a combination of qualitative, quantitative, and design methods. Of the 82 studies, 33 (40%) combined only qualitative methods with design methods. A detailed overview of the study characteristics is presented in Table 1.

**Table 1:** Characteristics of the included studies.

Author	Country	Title	End user population	Innovation type	Design approach	Study design
Bae et al [25]	Korea	Development of a user-centered health information service system for depressive symptom management	Patients who experience depression	Web-based system	User-centered system development	Mixed methods
Birnie et al [26]	Canada	ICanCope PostOp: user-centered design of a smartphone-based app for self-management of postoperative pain in children and adolescents	Children and adolescents who have recently undergone any type of day surgery	Pain self-management app	UCD^a^	Mixed methods
Brox et al [27]	Norway	User-centered design of serious games for older adults following 3 years of experience with exergames for seniors: a study design	Seniors	Serious game	UCD	Mixed methods
Cairns et al [28]	United Kingdom	Rethinking the foam cosmesis for people with lower limb absence	People with lower limb absence	Foam cosmesis for prosthetic limbs	User-centered product design	Mixed methods
Carey-Smith et al [29]	United Kingdom	A user-centered design process to develop technology to improve sleep quality in residential care homes	Older people with sleep or wake pattern disturbance	Sleep improvement technology	UCD	Qualitative
Caro et al [30]	Mexico	FroggyBobby: an exergame to support children with motor problems practicing motor coordination exercises during therapeutic interventions	Children with motor coordination problems	Exergames for children with motor problems	UCD	Qualitative
Catalani et al [31]	Kenya	A clinical decision support system for integrating tuberculosis and HIV care in Kenya: a human-centered design approach	HIV clinical care providers	Clinical shared decision support system	HCD^b^	Mixed methods
Cawood et al [32]	New Zealand	Creating the optimal workspace for hospital staff using human centered design	Hospital staff	Nonclinical workspaces	HCD	Qualitative
Civan-Hartzler et al [33]	United States	Bringing the field into focus: user-centered design of a patient expertise locator	Survivors of breast cancer	Patient expertise locator for web-based health communities	UCD	Qualitative
Connelly et al [34]	United States	Development of an ecological momentary assessment mobile app for a low-literacy, Mexican American population to collect disordered eating behaviors	Mexican-American women	Patient experiences assessment app	User-centered, iterative design	Mixed methods
Crespin et al [35]	Canada	Feasibility of adapting the fundamentals of laparoscopic surgery trainer box to endoscopic skills training tool	Surgeons and gastroenterologists	Laparoscopic surgery training box	UCD	Mixed methods
Curtis et al [36]	United Kingdom	Targeting parents for childhood weight management: development of a theory-driven and user-centered healthy eating app	Parents of children with weight management problems	Healthy eating app	UCD	Mixed methods
Dabbs de Vito et al [37]	United States	User-centered design and interactive health technologies for patients	Patients with a lung transplant	Personal health tracking app	UCD	Mixed methods
Das and Svanaes [38]	Norway	Human-centered methods in the design of an eHealth solution for patients undergoing weight loss treatment	Patients undergoing weight loss treatment	eHealth solution for weight loss treatment	HCD	Mixed methods
Davies et al [39]	United Kingdom	Recommendations for developing support tools with people suffering from chronic obstructive pulmonary disease: co-design and pilot testing of a mobile health prototype	People with COPD^c^	Mobile app for COPD self-management	User-centered, iterative design	Mixed methods
Dijkstra et al [40]	The Netherlands	Development of ehome, a mobile instrument for reporting, monitoring, and consulting drug-related problems in home care: human-centered design study	Home care nurses, general practitioners, and pharmacists	e-home solution for monitoring and consulting	HCD	Mixed methods
Eberhart et al [41]	United States	Using a human-centered design approach for collaborative decision-making in pediatric asthma care	Parents and children who are dealing with asthma management in a lower income environment	Physical decision-making aids	HCD	Qualitative
Erol Barkana and Açik [42]	Turkey	Improvement of design of a surgical interface using an eye tracking device	Surgeons who perform kidney tumor cryoablations	Eye-tracking device	UCD	Qualitative
Erwin et al [43]	United States	Development of a framework and tool to facilitate cost-of-care conversations with patients during prenatal care	Patients receiving prenatal care	Conversation framework	HCD	Qualitative
Ettinger et al [44]	South Africa	Building quality mHealth^d^ for low resource settings	Community health care workers	mHealth app to inform clinical decision-making	HCD	Mixed methods
Fabri et al [45]	United Kingdom	Using design thinking to engage autistic students in participatory design of an online toolkit to help with transition into higher education	Students with autism	Web-based toolkit	DT^e^	Mixed methods
Farinango et al [46]	Colombia	Human-centered design of a personal health record system for metabolic syndrome management based on the ISO 9241-210:2010 standard	Individuals at risk for metabolic syndrome	Personal health record system	HCD	Mixed methods
Ferris and Shepley [47]	United States	The design of neonatal incubators: a systems-oriented, human-centered approach	Infants, medical practitioners, and family members	Neonatal incubators	HCD	Qualitative
Foley et al [48]	United States	Primary care women’s health screening: a case study of a community engaged human centered design approach to enhancing the screening process	Women receiving health screening in primary care	Health screening tool	HCD	Qualitative
Fortuna et al [49]	United States	Adapting a psychosocial intervention for smartphone delivery to middle-aged and older adults with serious mental illness	Middle-aged and older adults with serious mental illnesses	Mobile app for medical and psychiatric self-management	UCD	Qualitative
Furberg et al [50]	United States	A digital decision support tool to enhance decisional capacity for clinical trial consent: design and development	People diagnosed with fragile X syndrome and clinicians	Tablet-based decision support tool	UCD	Qualitative
Gačnik et al [51]	Slovenia	User-centered app design for speech sound disorders interventions with tablet computers	Children with speech-language pathology	App for speech sound disorder therapy	UCD	Mixed methods
Garvelink et al [52]	Canada	Development of a decision guide to support the elderly in decision making about location of care: an iterative, user-centered design	Older adults and their informal caregivers	Decision guide (physical)	UCD	Mixed methods
Garvelink et al [53]	Canada	Deciding how to stay independent at home in later years: development and acceptability testing of an informative web-based module	Seniors with loss of autonomy	Interactive website	UCD	Qualitative
Garvin et al [54]	United States	Descriptive usability study of CirrODS: clinical decision and workflow support tool for management of patients with cirrhosis	Clinicians caring for patients with cirrhosis	Clinical decision and workflow support tool (digital)	UCD	Mixed methods
Garzo et al [55]	France	Design and development of a gait training system for Parkinson’s disease	People with Parkinson disease	Gait training app	UCD	Mixed methods
Gaynor et al [56]	United States	A user-centered, learning asthma smartphone application for patients and providers	People with asthma	Mobile app for asthma self-management	UCD	Qualitative
Gill et al [57]	Canada	Feasibility and acceptability of a mobile technology intervention to support post abortion care (The FACTS^f^ study phase II) after surgical abortion: user-centered design	Women who underwent an abortion	Web-based intervention for postabortion care support	UCD	Mixed methods
Giunti et al [58]	Spain	More stamina, a gamified mHealth solution for persons with multiple sclerosis: research through design	Young adults who have been diagnosed with multiple sclerosis	mHealth solution	UCD	Qualitative
Godinho et al [59]	Portugal	Improving accessibility of mobile devices with EasyWrite	Motor-disabled persons who experience text-entry difficulties when using mobile devices	Text-entry method for mobile devices	User-centered approach	Mixed methods
Gould et al [60]	United States	Development and refinement of educational materials to help older veterans use VA^g^ mental health mobile apps	Older veterans	Educational material for mobile mental health apps	UCD	Mixed methods
Green et al [61]	United States	Tracking care in the emergency department	Emergency department physicians	Emergency department tracking board	UCD	Qualitative
Griffin et al [62]	United States	Creating an mHealth app for colorectal cancer screening: user-centered design approach	People at risk for colorectal cancer aged ≥50 years	mHealth screening solution	UCD	Mixed methods
Grossman et al [63]	United States	Leveraging patient-reported outcomes using data visualization	Patients with heart failure and health care providers for patients with heart failure	Data visualization	UCD	Mixed methods
Hafiz et al [64]	Denmark	The internet-based cognitive assessment tool: system design and feasibility study	Patients with unipolar and bipolar disorder	Web-based cognitive assessment tool	UCD	Mixed methods
Hardy et al [65]	United Kingdom	How inclusive, user-centered design research can improve psychological therapies for psychosis: development of SlowMo	People who fear harm from others	Digital solution for psychological therapy	UCD	Qualitative
Harte, R. [66]	Ireland	Human-centered design study: enhancing the usability of a mobile phone app in an integrated falls risk detection system for use by older adult users	Older adults with fall risk	Mobile app for fall risk detection	HCD	Mixed methods
Hartlzer et al [67]	United States	Design and feasibility of integrating personalized PRO^h^ dashboards into prostate cancer care	Patients following prostate cancer treatment	Patient dashboard	HCD	Mixed methods
Herschman et al [68]	Canada	Development of a smartphone app for adolescents with lupus: a collaborative meeting-based methodology inclusive of a wide range of stakeholders	Adolescents with lupus	Mobile app for adolescents	UCD	N/A^i^
Horsky and Ramelson [69]	United States	Development of a cognitive framework of patient record summary review in the formative phase of user-centered design	Clinicians	Patient record summary review	UCD	Qualitative
Huberty et al [70]	United States	Development and design of an intervention to improve physical activity in pregnant women using Text4baby	Pregnant women	SMS text messaging	UCD	Mixed methods
Isenberg et al [71]	United States	An advance care plan decision support video before major surgery: a patient- and family-centered approach	Patients who are preparing for major surgery	Advance care planning decision support video	HCD	Mixed methods
Johnston et al [72]	United States	Designing and testing a web-based interface for self-monitoring of exercise and symptoms for older adults with COPD	Older adults with COPD	Web-based interface for self-monitoring of exercise	UCD	Mixed methods
Lan Hing Ting et al [73]	France	Examining usage to ensure utility: co-design of a tool for fall prevention	Older adults with fall risk	Balance assessment tool	HCD	Mixed methods
Luna et al [74]	Argentina	User-centered design improves the usability of drug-drug interaction alerts: experimental comparison of interfaces	Physicians	Drug–drug interaction alert system	UCD	Mixed methods
Ma, Wu and Chang [75]	Taiwan	A new design approach of user-centered design on a personal assistive bathing device for hemiplegia	Patients with stroke and hemiplegia	Personal assistive bathing device	UCD	Qualitative
Madrigal-Cadavid et al [76]	Colombia	Design and development of a mobile app of drug information for people with visual impairment	People with visual impairment	Mobile app for drug information	UCD	Qualitative
Marker and Monzon [77]	United States	Iterative development of a web-based intervention for families of young children with type 1 diabetes: DIPPer academy	Parents of children with type 1 diabetes	Web-based intervention	UCD	Mixed methods
Marko-Holguin et al [78]	United States	A two-way interactive text messaging application for low-income patients with chronic medical conditions: design-thinking development approach	Patients with low income and chronic medical conditions	Interactive SMS text messaging app	DT	Mixed methods
Martin et al [79]	Ireland	A qualitative study adopting a user-centered approach to design and validate a brain computer interface for cognitive rehabilitation for people with brain injury	People with brain injury	Brain-computer interface	UCD	Qualitative
McGinn et al [80]	Ireland	A human-oriented framework for developing assistive service robots	People with disabilities	Assistive service robot	HCD	Qualitative
McMullen et al [81]	United States	Designing for impact: identifying stakeholder-driven interventions to support recovery after major cancer surgery	Patients who recover from major cancer surgery	Web-based educational platform for patients	UCD	Qualitative
Melnick et al [82]	United States	Patient-centered decision support: formative usability evaluation of integrated clinical decision support with a patient decision aid for minor head injury in the emergency department	Emergency department physicians	Electronic clinical decision support	UCD	Mixed methods
Nunez-Nava et al [83]	Colombia	Human-centered development of an online social network for metabolic syndrome management	People with metabolic syndrome	Web-based social network	HCD	Mixed methods
Person et al [84]	Tanzania	Community co-designed schistosomiasis control interventions for school-aged children in Zanzibar	School-aged children	Intervention to reduce schistosomiasis transmission	HCD	Qualitative
Petersen, and Hempler [85]	Denmark	Development and testing of a mobile application to support diabetes self-management for people with newly diagnosed type 2 diabetes: a design thinking case study	People with newly diagnosed type 2 diabetes	Mobile app for newly diagnosed patients with type 2 diabetes	DT	Qualitative
Ragouzeos et al [86]	United States	*Am I OK?* using human centered design to empower rheumatoid arthritis patients through patient reported outcomes	Patient with rheumatoid arthritis	Dashboard to display PROs	HCD	Qualitative
Ray et al [87]	United States	Computerized clinical decision support system for emergency department–initiated buprenorphine for opioid use disorder: user-centered design	Emergency department physicians	Computerized clinical decision support system	UCD	Qualitative
Rothgangel et al [88]	The Netherlands	Design and development of a telerehabilitation platform for patients with phantom limb pain: a user-centered approach	Patients with phantom limb pain	Tele-rehabilitation platform	UCD	Mixed methods
Salmon et al [89]	Congo	Alternative ultrasound gel for a sustainable ultrasound program: application of human centered design	Local clinicians who use point of care ultrasound	Alternative ultrasound gel	HCD	Mixed methods
Schild et al [90]	Germany	A digital cognitive aid for anesthesia to support intraoperative crisis management: results of the user-centered design process	Anesthesiologists	Digital cognitive aid for intraoperative crisis management	UCD	Mixed methods
Sedlmayr et al [91]	Germany	User-centered design of a mobile medication management	People who use medication	Mobile interface for medication management	UCD	Mixed methods
Seeber et al [92]	Germany	A design thinking approach to effective vaccine safety communication	Parents and babies	Effective vaccine safety communication	DT	Qualitative
Sonney et al [93]	United States	Applying human-centered design to the development of an asthma essentials kit for school aged children and their parents	School-aged children and their parents who deal with asthma management	Asthma essential kit	HCD	Qualitative
Srinivas et al [94]	United States	Context-sensitive ecologic momentary assessment: application of user-centered design for improving user satisfaction and engagement during self-report	Middle-aged women with obesity	Patients’ experiences assessment app	UCD	Mixed methods
Stevens et al [95]	The Netherlands	The development of a patient-specific method for physiotherapy goal setting: a user-centered design	Physiotherapists and patients	A new method for goal setting	UCD	Qualitative
Taylor et al [96]	United States	User-centered development of a web-based preschool vision screening tool	Parents of preschool-aged children with amblyopia	Web-based vision screening tool	UCD	Mixed methods
Timmerman et al [97]	The Netherlands	Cocreation of an ICT^j^-supported cancer rehabilitation application for resected lung cancer survivors: design and evaluation	Health care professionals and survivors of lung cancer	ICT-supported cancer rehabilitation program	UCD	Mixed methods
Tucker Edmonds et al [98]	United States	Creation of a decision support tool for expectant parents facing threatened periviable delivery: application of a user-centered design approach	Prospective parents	Decision support tool	UCD	N/A
van der Weegen et al [99]	The Netherlands	The development of a mobile monitoring and feedback tool to stimulate physical activity of people with a chronic disease in primary care: a user-centered design	People with chronic disease	Mobile monitoring and feedback tool	UCD	Qualitative
Vechakul et al [100]	United States	Human-centered design as an approach for place-based innovation in public health: a case study from Oakland, California	Citizens of Castlemont neighborhood	Novel programs to reduce inequities in infant mortality rates	HCD	Qualitative
Vermeulen et al [101]	The Netherlands	User-centered development and testing of a monitoring system that provides feedback regarding physical functioning to elderly people	Older adults	Mobile interface for a monitoring system	User-centered development process	Mixed methods
Vilardaga et al [102]	United States	User-centered design of learn to quit, a smoking cessation smartphone app for people with serious mental illness	People with serious mental illnesses who smoke	Smoking cessation app	UCD	Mixed methods
Wachtler et al [103]	Australia	Development of a mobile clinical prediction tool to estimate future depression severity and guide treatment in primary care: user-centered design	People with depressive symptoms	App for improvement of treatment allocation for depression	UCD	Qualitative
Willard et al [104]	The Netherlands	Development and testing of an online community care platform for frail older adults in The Netherlands: a user-centered design	Frail older adults	Web-based community platform	UCD	Mixed methods
Woodard et al [105]	United States	The Pathways fertility preservation decision aid website for women with cancer: development and field testing	Women survivors of cancer	Decision aid website for young women with cancer	UCD	Mixed methods
Wysocki et al [106]	United States	A web-based coping intervention by and for parents of very young children with type 1 diabetes: user-centered design	Parents of young children with type 1 diabetes	Web-based coping resource	UCD	Qualitative

^a^UCD: user-centered design.

^b^HCD: human-centered design.

^c^COPD: chronic obstructive pulmonary disease.

^d^mHealth: mobile health.

^e^DT: design thinking.

^f^FACTS: factors affecting combination trial success.

^g^VA: veterans affairs.

^h^PRO: patient-reported outcome.

^i^N/A: not applicable.

^j^ICT: information and communication technology.

### Design Theories and Methodologies

This review explores the various applications of HCD approaches, including HCD, UCD, and DT. Of the 82 studies, HCD was used in 21 (26%) studies, whereas 4 (4%) studies applied a DT approach. Most (57/82, 70%) used a UCD approach. All approaches prioritized the users’ needs and the participatory and iterative nature of the design process. Some HCD definitions included a focus on a multiple stakeholder or system perspective, whereas some UCD definitions aimed at increasing usability or user friendliness of the solution. These design approaches are generally characterized by the use of different standards or models.

**Figure 2:**
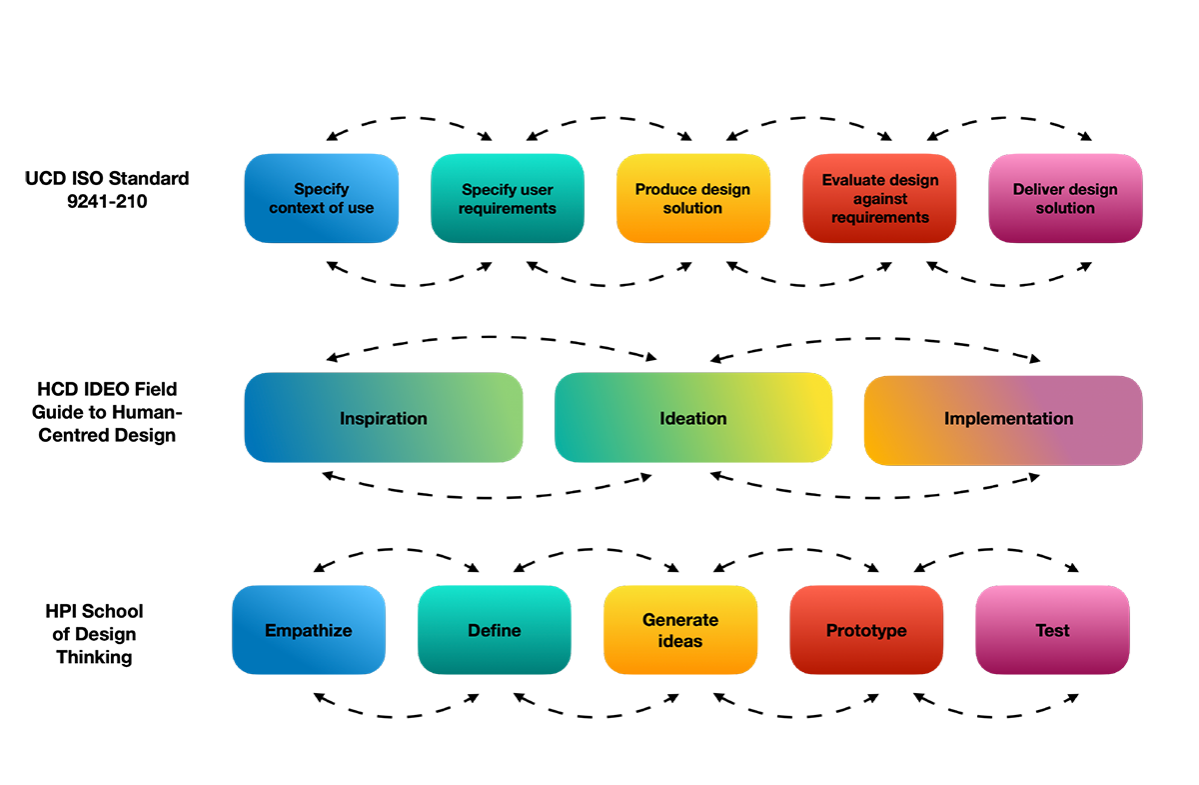
Illustration of human-centered design processes. HCD: human-centered design; HPI: Hasso Plattner Institute; UCD: user-centered design.

A total of 3 standards or models were frequently mentioned in the studies and used as references. These models overlap in their attempt to classify the distinct phases of the design process but operationalize the steps differently. The UCD ISO Standard 9241-210 for HCD of interactive systems encompasses a 5-phase design process including (1) understanding and specifying the context of use, (2) specifying user requirements, (3) producing design solutions, (4) evaluating design against requirements, and (5) delivering design solutions that meet user requirements. The HCD IDEO Field Guide to Human-Centered Design and the Hasso Plattner Institute (HPI) School of Design Thinking models are characterized by different versions of a similar 3-phase design process: (1) inspiration, (2) ideation, and (3) implementation. Studies that applied DT worked with a multiphase approach that included versions of the following phases: (1) empathizing with stakeholders, (2) defining the problem, (3) generating ideas for solutions, (4) prototyping the solutions, and (5) testing the solutions. In Figure 2, we have

illustrated how the different approaches to the HCD process align.

Of the 82 articles identified, 57 (70%) applied a UCD approach, 21 (26%) used HCD, and 4 (5%) used DT. In 17% (14/82) of the studies, the concepts of HCD and UCD were referred to interchangeably; of these 14 studies, 9 (64%) studies referred to the use of the ISO 9241-210 standard. In the 5% (4/82) of studies that applied DT, the concept was used interchangeably with HCD in all cases. These studies referred to the IDEO Field Guide to Human-Centered Design or the HPI School of Design Thinking Guide as standards. For clarity, we have continued to report the results of the HCD and DT studies and UCD studies separately in this review.

### Design Strategies and Methods

Of the 82 studies, 74 (90%) applied an SFS versus 8 (10%) applied a PFS to drive the design process. Thus, most design studies focused on directly generating solutions or developing a specific predefined solution. Only a minority used design-based methods to define the problem and selectively gather information before proceeding to solution development. Of the 74 studies that applied an SFS, 55 (74%) applied the UCD approach. Of the 8 studies that applied a PFS, 6 (75%) applied an HCD and DT approach. Overall, HCD and DT appears to be the preferred approach for problem-driven strategies, whereas UCD is generally applied for solution-driven strategies.

The design processes comprised several design cycles during which multiple qualitative and quantitative methods were used in combination with specific design methods. Of the 82 studies, 47 (57%) applied a mixed methods approach, and 33 (40%) applied qualitative methodology. A synthesis of the methods used in the different phases of the included studies is presented in Table 2 (details about the described design methods can be found in Multimedia Appendix 3). The first design phase—understanding the context—was often characterized by the use of a limited range of design-based methods. During the second and third phases—problem specification and idea generation—a broader range of design methods was used in different studies. In the fourth phase—testing of solutions—the range of design methods was reduced again. Some design-based methods were applied in multiple phases of the process, for example, personas, intervention mapping, or the Wizard of Oz technique; however, most were uniquely used in a single phase.

Overall, qualitative methods or mixed methods were mostly used in the first and last phases of the design process to understand user needs or to evaluate user experiences. In the first phase of the process, qualitative methods such as interviews and observations as well as literature reviews were commonly used to understand the problem context. In later stages, the use of methods diverges based on the type of foreseen solution, for example, digital or nondigital solutions. Quantitative methods were used to either support qualitative findings during the first phase of the process or as an evaluation instrument in the later design phases.

**Table 2:** Meta-analysis of applied research and design methods.

Design phase	Qualitative methods	Quantitative methods	Design methods
Understanding the context	Literature review Observations Expert meetings Delphi technique Diary studies	Surveys (not specified)	Storytelling Metaphors Personas Experience mapping
Specify the problem or user need	Focus groups Interviews Delphi technique Contextual inquiry Observations Critical incident technique	Context assessments Needs assessments Surveys (not specified)	Participatory workshop Personas Use case scenarios Decision matrix MoSCoW^a^ method House of quality analysis Goal, question, metric approach Roleplay User journey mapping Intervention mapping System mapping Low functional prototype Use case diagram
Generate ideas and design solutions	Observations Interviews Focus groups Literature review	Usability surveys Feasibility surveys Surveys (not specified)	Brainstorm Round Robin Concept Ideation Voting Round table discussions Sketching Visual mind maps Idea or concept voting Storyboarding User narratives Use case scenarios Low functional prototyping High functional prototyping Intervention mapping Heuristic evaluation Task analysis SWOT^b^ or competitor analysis User journey map Wizard of Oz method Card sorting Weekly sprints Think-aloud techniques
Test solutions	Interviews Observations Focus groups EMA^c^	Usability surveys Feasibility surveys Viability assessments EMA Surveys (not specified)	Low functional prototyping High functional prototyping Roleplay Story boarding Card sorting Simulations Intervention mapping Cognitive walkthrough Brainstorm (general) Heuristic evaluation Workflow evaluation Participatory workshop Wizard of Oz method Value versus effort matrix Think-aloud techniques

^a^MoSCoW: must have, should have, could have, won’t have.

^b^SWOT: strengths, weaknesses, opportunities, and threats.

^c^EMA: ecological momentary assessment.

### End User Involvement

**Figure 3:**
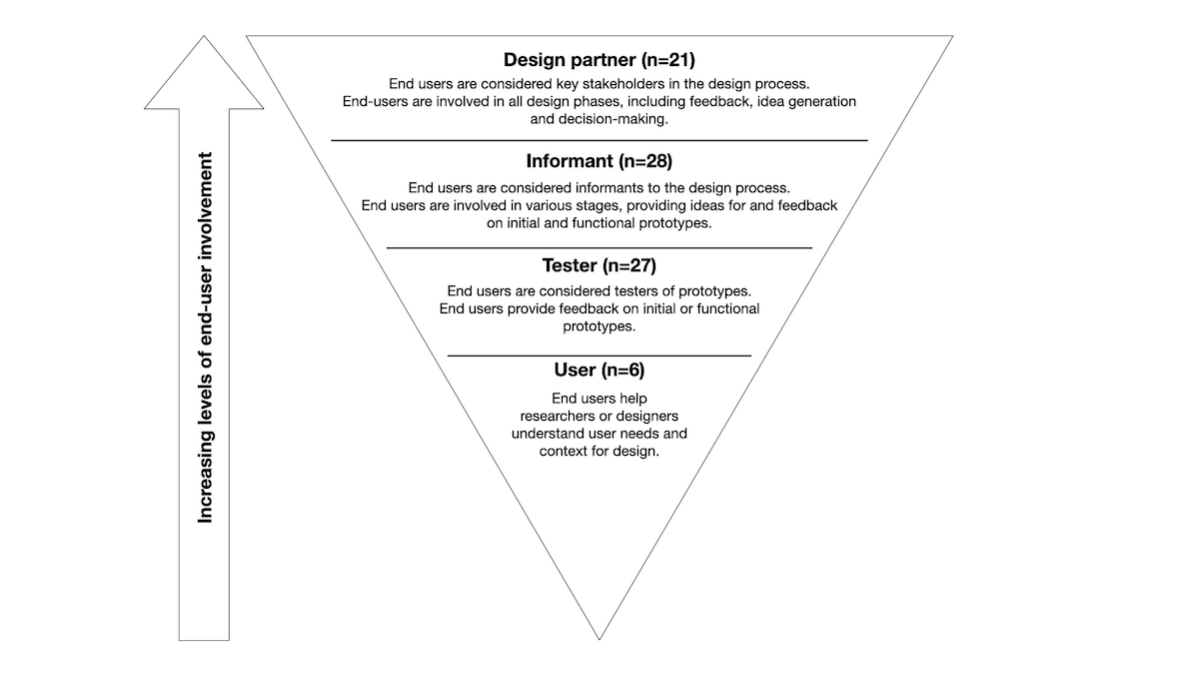
Levels of end user involvement during human-centered design processes.

In 6 studies (UCD 5/6, 83%; HCD and DT 1/6, 17%), the end users were actively involved as *users*, that is, as information sources but not as active participants in the design process. In 27 (UCD 21/27, 78%; HCD and DT 6/27, 22%) studies, the end users participated as *testers*; that is, they were involved in the first and last phases of the design process as testers of the developed solutions. In 28 (UCD 22/28, 79%; HCT and DT 6/28, 21%) studies, the end users were involved as *informants*. Here, end users were involved in various phases of the process and asked for input on the design prototypes, such as sketches and low-fidelity prototypes. Participation as *design partners*, that is, as contributors to all phases and being involved in the decision-making process, was identified in 21 (UCD 9/21, 43%; HCD and DT 12/21, 57%) studies (Figure 3). Although UCD approaches involved end users primarily in the role of tester (21/57, 37%) and informant (22/57, 39%), HCD and DT approaches involved end users as design partners in 48% (12/57) of the studies.

### Quality Assessment of the Studies

Using the MMAT, 16% (13/82) of the included studies met ≥1 MMAT reporting criteria, based on the study type. The remaining studies had to be rated as unclear on all MMAT reporting criteria. An overview of the quality assessment results can be found in Multimedia Appendix 4 [25-106]. The biggest limitation to the quality assessment was the lack of uniformity in reporting and the broad extent of the design studies that needed to be captured in limited words for publication. In fact, most studies used multiple research and design cycles and generally offered limited details about the applied methodology.

## Discussion

### Principal Findings

In this review, we explored how different HCD approaches, including DT and UCD, were applied for the development of innovations in health research. Overall, the concepts of HCD and DT, and HCD and UCD, were used interchangeably in 22% (18/82) of the included studies. This applied to all studies that referred to HCD and DT; however, UCD was defined as a standalone entity in 84% (48/57) of the papers that used this approach. Most of the studies using HCD and UCD interchangeably referred to the ISO 9241-210 standard. This aligns with the theoretical framework pursued by the studies, that is, a problem-driven versus a solution-driven strategy. DT- and HCD-based studies commonly engaged in understanding the underlying problem and focused on a broad range of health, social, or medical topics. They often included a focus on human values and a multistakeholder or systems perspective. Instead, UCD-based approaches focused primarily on the direct identification of a solution and were mostly used in health technology innovation. They often focused on human factors to increase the usability or user friendliness of the solution. The limitations of this functional approach in promoting human interests have been previously described as a potential shortcoming of UCD [107].

It has been reported that designers who use a problem-driven design strategy produce solutions with the best balance between quality and creativity [22]. However, in this review, 90% (74/82) of the included studies used a solution-driven strategy. Although the evaluation of solutions can be used to further define the design problem, this was not an objective of the included studies. Their solution-driven approach generally focused on generating a large number of ideas and solutions, potentially leaving the initial design problem ill-defined and ignoring the relationships between various stakeholders. However, health care innovation could significantly benefit from problem-driven design processes, especially from the perspective of resource efficiency. Innovation in health care is characterized by a development or implementation cost trade-off. Therefore, it is critical that the most impactful innovations be prioritized based on a critical understanding of the underlying problem [108].

HCD in health research is often perceived as a single unitary method, as emphasized by the reference to a single practitioner guideline in the included studies. However, in this review, we found that the application of HCD entails a wide array of design methods and techniques that can be used selectively and that are dependent on the specific design case. Design methods diverge from the traditional methods of academic research as they are primarily oriented toward action or solution of defined problems rather than toward theory and hypotheses building. To date, little is known about their effectiveness according to evidence-based medical standards. The creation of a new product, system, or service to improve health might be considered an outcome from a design perspective but would not be considered a health outcome from a scientific perspective [13,14]. In the literature, a scientific method is described as a strategy to understand the nature of a phenomenon, whereas a design method is a strategy to invent things of value. According to this distinction, science is analytical and design is constructive and it is therefore difficult to assess both methods according to the same standard [20]. However, according to Frey and Dym [109], many of the validation techniques found in medicine can be used for the validation of design methods. For example, where medicine uses animal models and clinical trials to test medical treatments, detailed simulations and controlled field experiments of design methods could be developed for the explicit purpose of evaluating design methodologies [109].

This logical, empirical approach toward the evaluation of design methods fits well with, for example, the field of engineering design, which is based on mathematical modeling, as it is most appropriate for closed, objective problems that can lead to binary (yes or no) answers. However, HCD approaches often address open, complex problems that involve both objective and subjective elements without a single *correct* answer. For design methods addressing open, complex problems, a relativist validation approach that gradually builds confidence in the usefulness of the methods can be considered a more appropriate paradigm [110]. A relativist approach to design claims no absolute objectivity for methods or models; however, it assumes that a valid method or model is only one of the many possible ways of measuring or describing a real situation. In a relativist approach to design methods, validity becomes a matter of practical use and contextual functionality rather than formal and universal accuracy. The validity of design methods becomes a contextual, semiformal, and conversational process, because establishing models of usefulness is a conversational matter [111]. It is important to note that a relativist approach toward the evaluation of design methods does not antagonize the logical, empirical approach toward the evaluation of scientific research methods used in HCD processes.

There is an ongoing demand for the development of a *design science* with systematic and formalized design methods that adhere to the values of the empirical scientific method: objectivity, rationality, and universalism [112,113]. Scientific design methods have been developed in engineering and computer science; however, there is limited evidence that the systematic use of design practices leads to measurable and reproducible results in health research [112]. Design researchers themselves still debate whether design conforms to a scientific activity or represents an academic discipline with a rigorous culture of its own [20,113]. As a result, critical appraisal and best practice selections of design methods in health research remain challenging.

In this review, the diverse reporting formats challenged our ability to assess the quality of the studies from an evidence-based perspective. Although initial guidelines have been proposed to improve the reporting of design studies in health research, this is still an area that is in development [14]. The guidelines by Bazzano et al [14] represent the first detailed overview of reporting items for health research that includes design approaches. Although we acknowledge that this reporting guideline is an important first step toward improving transparency, evaluability, and wider dissemination of design approaches in health research, it is, however, debatable whether the application of these guidelines is feasible in the context of health research manuscripts. The level of detail that the Bazzano [14] guidelines propose implies that the design research component should be reported as a standalone article, separate from the connected empirical studies. Most of the design studies included in this review offered limited details about their multimethod design cycles, possibly because of the word count limits that most scientific journals apply. It would be almost impossible to describe a multimethod design process in adequate detail and also effectively report on the research and design outcomes in a single manuscript. Applying the Bazzano [14] guidelines with rigor is likely to result in the reporting of separate design cycles across multiple manuscripts, and essential findings for the design process might appear fragmented or be lost among reports that are published separately.

However, it could be argued that the separate publication of multiple waves of data collection in design research is preferable for both researchers and reviewers to support the validity, reliability, and reproducibility of design-based health research. Rather than aiming for complex integrated manuscripts, multiple publications would allow researchers to report in more detail on both their methods and findings and also allow for easier critical appraisal and quality assessment by reviewers. In addition to traditional research articles, innovative publication formats such as registered reports could be used to submit design research protocols and results that are judged on their methodological robustness rather than the potential novelty of the findings [114]. We recommend registering the design research protocols in a research registry to address the issue of potential fragmented data publication. This would allow for systematic referencing to previous design activities, even when their results have not been published.

The active engagement of stakeholders is one of the key principles of the HCD approach. Stakeholders can be defined as “individuals, organizations or communities that have a direct interest in the process and outcomes of a project, research or policy endeavor” [115]. In health care innovation, the engagement of diverse stakeholders is essential to the development of a shared agenda for responsible innovation and for the cocreation of social value [116]. However, a multistakeholder innovation process brings about several challenges. HCD practitioners acknowledge the challenge of equitably including the experience and expertise of all participants in the design process. Although the importance of creative interdisciplinary collaborations between various disciplines in health care is increasing, it is still a relatively new and complex phenomenon [117]. Each stakeholder brings their own motivations, attitudes, priorities, and incentives to the process, and such differences will influence the cocreative space and interpersonal interactions. HCD practitioners should critically reflect on the participatory methods that they intend to apply, considering the possible contribution of each participant in the design process to facilitate the effective use of their expertise and experiences [16]. This is particularly important when working with vulnerable patient groups or health care professionals with limited time to participate in co-design sessions [118].

An earlier study suggested that HCD processes can rely too much on anecdotal evidence of key stakeholders who might not fully understand what they want and need [31]. However, a more strategic application of HCD aims to identify themes that describe people’s deeper needs and values rather than their wishes and desires and uses those themes to inform the creation of innovative strategies and solutions [119]. Field studies with the use of qualitative methods, such as observations, to study key stakeholders and their activities in their own environments could offer a valid alternative [120].

In addition, it is essential for HCD practitioners to take power dimensions and the agency of different stakeholders into account, especially during co-design sessions. To achieve inclusive design processes, intersectional aspects should be considered for stakeholder engagement and methodological choices, such as gender identity, class, sexuality, geography, age, and disability and ability [121,122]. Reflective project planning aids and frameworks for involving patients and the public in research and design projects should be used to guarantee meaningful engagement of stakeholders and facilitate democratic design processes [123,124].

### Study Limitations

At present, MMAT is the most comprehensive tool available for appraising multimethod studies [125]. Although the MMAT is a tool that allows for the critical appraisal of most common types of study designs, the tool seems less appropriate for HCD, DT, and UCD because of the inclusion of multiple research and design cycles and the often-limited word space to describe the applied methodologies and methodological choices in detail. To our knowledge, there is no appropriate tool available for the critical appraisal of design studies in health research.

In this review, we have only reviewed articles that described the complete development processes of a health innovation. This criterion might have limited the inclusion of studies that describe the complete process through multiple publications. For example, in a few studies, the authors referred to future studies in which they expressed the intention to test a designed solution in a randomized controlled trial. Those studies were not included in this review. Furthermore, no selection criteria for the end user populations were applied. This might have influenced the choice for the use of particular design methods, as design researchers need to take intersectional aspects into account, as mentioned in the *Discussion*. Although this was not the main objective of this review, future research could focus on the application of design methods and their suitability for specific stakeholder populations in health care.

In addition, our search strategy was limited to scientific databases related to biomedical, nursing, and allied health and public health sciences, and gray literature was not included. Disciplines that publish design research related to health systems outside this scope were not considered in our searches. Finally, the existence of different design methods and models with principles related to HCD and the interchangeable use of these terms in the literature made it challenging to scope and perform a fully systematic search.

### Conclusions

A wide variety of design practices and methods such as HCD, DT, and UCD are increasingly being applied in health research. In our analysis, HCD- and DT-based projects tended to primarily follow integrated and problem-driven approaches, whereas UCD-based projects engaged in more functional and solution-driven approaches. Most of these design studies used mixed methods approaches, combined qualitative and quantitative research with design methods, and frequently referred to the following 3 design guides: the IDEO Field Guide to Human-Centered Design, the HPI School of Design Thinking Guide, and the ISO Standard 9241-210.

The increasing use of design-based approaches such as HCD and DT and UCD in health research subjects them to evaluation according to traditional biomedical standards. However, the analytic approach of the scientific method versus the constructive approach of the design method impedes the assessment of both methods according to the same standard. To address the validation of design methods, a relativist validation approach that gradually builds confidence in the usefulness of methods could be considered a more appropriate paradigm for design methods, particularly those that are concerned with subjective elements of the design process.

Specific standards for reporting HCD practices in health and biomedical research have been developed in recent years. However, these reporting standards remain challenging to apply for single design research papers because of the extensiveness of multimethod design processes in combination with customary word limits in biomedical publications. Separate publications detailing the multiple waves of data collection in design research might be preferable for both researchers and reviewers to support the validity, reliability, and reproducibility of design-based health research. In addition, innovative publication formats such as registered reports could be used to submit design research protocols and results that are judged on their methodological robustness rather than the potential novelty of the findings. Furthermore, future research on HCD approaches in health should focus on the development of an HCD practitioner guideline for stakeholder engagement that takes stakeholder roles, experiences, expertise, agency, and power dimensions into account.

## Appendix

Multimedia Appendix 1 Review protocol.

Multimedia Appendix 2 Search strategy.

Multimedia Appendix 3 Descriptions of the design method included in the review.

Multimedia Appendix 4 Mixed Methods Appraisal Tool checklist.

## Abbreviations

DT: design thinking
HCD: human-centered design
HPI: Hasso Plattner Institute
MMAT: Mixed Methods Appraisal Tool
PFS: problem-focused strategy
SFS: solution-focused strategy
UCD: user-centered design
