# Supplementary material for: The Application of Human-Centered Design Approaches in Health Research and Innovation: A Narrative Review of Current Practices
Source: JMIR Mhealth Uhealth. 2021 Dec 6;9(12):e28102. doi: 10.2196/28102 (PMC8691403; doi:10.2196/28102)
Supplement: Multimedia Appendix 1 [file mhealth_v9i12e28102_app1.doc]

### Supplement: Systematic Narrative Review Protocol

### Research Title

The application of human-centered design in healthcare

### Review Question

1. How is the human-centered design (HCD) process applied in design research in the field of healthcare?

2. What is the reporting quality of these studies?

3. Which design methods are used in the HCD process?

4. How are stakeholder involved during the design process?

### Types of studies to be included

Development studies, Qualitative studies, Mixed-method studies, Case-control studies, Cohort studies, RCT’s Experimental studies, Cross-sectional studies, and any other deemed appropriate for inclusion will be included.

Editorials, opinion papers, reviews, animal studies, lab studies and meta-analyses will be excluded.

### Database Searches

The appropriate databases will be selected in collaboration with librarian experts in medical sciences, social sciences and business sciences. The following databases will be searched: Pubmed, CINAHL, Embase, The Cochcrane Library, PsycInfo, Web of Science and Sociological Abstracts. Articles published between 2000 and 2019 and written in the English language will be eligible for inclusion.

**Search Strategy**

Keyword search for Pubmed, Cinahl, Embase and the Cochcrane Library databases:

*(((human-centered OR human-centred) OR (user-centered OR user-centred)) AND (design OR approach)) OR design thinking*

Keyword search for PsycInfo, Web of Science and Sociological Abstract databases:

*(((human-centered OR human-centred) OR (user-centered OR user-centred)) AND (design OR approach)) OR design thinking AND (health* OR medic* OR clinic*)*

**Eligibility Criteria**

Study characteristic for inclusion are studies that 1) applied HCD/DT/UCD, 2) focus on the development of health innovations and 3) provide a detailed description of the design process, including: 4) the applied process steps and/or phases, 5) the applied design methods per process step and/or phase and 6) a description of the involved design team and stakeholders.

In order to frame the concept of ‘health innovation’, a modified definition of the WHO will be used: *“Health innovation identifies new or improved health policies, systems, products and technologies, and services and delivery methods that improve people’s health and wellbeing.”*

Studies will be excluded if: 1) the study itself does not focus on the design process and 2) the study does not provide a detailed description of the design process and design methods.

### Condition or domain being studied

The aim of this studies is to conduct a systematic review on the application of human-centered design in the context of healthcare.

### Participant/population

All participants will be included as long as they participated as either a designer or stakeholder in the design process in a study featuring the application of human-centered design.

### Intervention(s), exposure(s)

Design or development studies of health innovations that aim to improve health policies, systems, products, technology and services or healthcare delivery methods that improve people’s health and wellbeing will be included.

### Comparator(s)/control

The comparator is the target user before and after they have been exposed to an intervention created through a human-centered design process. The comparator is only applicable for HCD studies that evaluate the effectiveness of the intervention or development process. However, we do not expect to find information about the effectiveness of the intervention or development process.

### Context

The development of health innovation in the medical setting.

### Main outcome(s)

We expect that this review will contribute to a better understanding of the human-centered design process as it is applied in health innovation studies and will provide an overview of the design methods that are used in these design processes.

### Additional outcome(s)

Additional outcomes for the study include analyzing the engagement of end-users / stakeholders in the design process.

### Data extraction (selection and coding)

The studies collected from the selected databases will be downloaded into a reference manager and de-duplicated. The de-duplicated results will then be uploaded into the screening program Rayyan for the first screening based on titles and abstracts. The first screening procedure will be performed by two reviewers independently, based on the first, second and (likelihood of the) third eligibility criteria. Excluded articles will be labeled according to the predominant exclusion criterium that they were excluded on. In case of disagreement, consensus will be achieved by discussion among the reviews or consultation of a third party. In order to determine the level of agreement, the Cohen’s K and percentage of agreement will be calculated.

The second screening will involve full-text screening of the included articles and will follow the same procedure as the first screening, including all eligibility criteria.

When reading the full texts of included articles, the following characteristics will be extracted:

First author, title, year of publication, aim of the study, location of the study, study design, end-user of the design, type of innovation, design approach, design approach referent.

### Risk of bias (quality) assessment

Bias risk evaluation will be performed using the Joanna Briggs Institute Critical Appraisal Tools. Mixed method studies will be assessed using the Mixed Methods Appraisal Tool (MMAT).

### Strategy for data synthesis

Data extraction will be performed by items and will differ per search strategy.

The first strategy will focus on the analysis of the study characteristic, leading to the formation of a baseline table.

The second strategy will focus on the analysis of quality assessment of the study, study design and applied design methods, leading to a summary of the findings.

The third strategy will focus on the analysis of the level of engagement of the stakeholder in the design process, leading to a summary of the findings.

### Analysis of subgroups or subsets

No analysis of subgroups or subsets will be performed.

### Contact details for further information

Irene Göttgens ([irene.gottgens@radboudumc.nl](mailto:irene.gottgens@radboudumc.nl))

### Organisation affiliation of the review

Radboud University Medical Center.

### Review team members and their organizational affiliation

Prof. dr. Sabine Oertelt-Prigione, Radboud University Medical Center

### Type and method of review

Systematic review, narrative synthesis

### Anticipated or actual start date

1 July 2019

### Anticipated completion date

31 December 2020

### Funding sources/sponsors

Infrastructure and research resources, such as library material, database subscriptions, analysis software and working infrastructure, will be provided by the Radboud University Medical Center.

No further funding is acquired for this study.

### Conflict of interest

None known

### Language

English

### Country

The Netherlands

### Stage of review

Review ongoing

### Subject index term status

Unknown

### Subject index terms

Human-centered design, Health innovation, design methods

### Date of registration in Prospero

Unknown

### Date of publication of this version

Unknown

### Revision notes for this version

None

### Stage of review at the time of this submission

**Stage Started Completed**

Preliminary searches Yes Yes

Piloting selection process Yes Yes

Formal screening of search results against eligibility criteria Yes No

Data extraction No No

Risk of bias (quality) assessment No No

Data analysis No No
