# Supplementary material for: The Application of Human-Centered Design Approaches in Health Research and Innovation: A Narrative Review of Current Practices
Source: JMIR Mhealth Uhealth. 2021 Dec 6;9(12):e28102. doi: 10.2196/28102 (PMC8691403; doi:10.2196/28102)
Supplement: Multimedia Appendix 2 [file mhealth_v9i12e28102_app2.doc]

**Supplement: Search strategies and results**

**SEARCH 07.2019**

**Medical databases:**

**Search strategy:** *((human-cent* OR user-cent*) AND (design OR approach)) OR design thinking*

Limitations: Title, Abstract, Published between 01-2000 and present, English Language

**Database: Number of articles found:**

Pubmed: 1443

Embase: 1759

Cinahl: 830

Cochrane: 145

**Non-medical databases:**

**Search strategy:** *(((human-cent* OR user-cent*) AND (design OR approach)) OR design thinking) AND (health* OR medic* OR clinic*)*

Limitations: Title, Abstract, Heading word/All subjects & indexing (SU)Published between 01-2000 and present, English Language

**Database: Number of articles found:**

PsycInfo: 249

Web of Science: 1309

Sociological Abstracts: 22

Subtotal: 5757

Duplicates: 2817

Total references for first screening: **2940**

**UPDATED SEARCH 08.2020**

**Medical Database: Number of articles found:**

Pubmed: 424

Embase: 537

Cinahl: 283

Cochrane: 50

**Non-medical Database: Number of articles found:**

PsycInfo: 46

Web of Science: 460

Sociological Abstracts: 3

Subtotal: 1803

Duplicates: 1255

Total references for updated screening: **548**

**Total references first + updated screening 3488**

**Search Strategy by Database**

**PUBMED:**

((human-cent*[tiab] OR user-cent*[tiab]) AND (design[tiab] OR approach[tiab]) OR design thinking[tiab]

**Embase:**

(((human-cent* or user-cent*) and (design or approach) or design thinking).ti,ab,kw,hw

**Cinahl**

(((TI (human-cent* OR user-cent*) OR AB (human-cent* OR user-cent*) OR SU (human-cent* OR user-cent*)) AND (TI (design OR approach) OR AB (design OR approach) OR SU (design OR

approach)) OR (TI design-thinking OR AB design-thinking OR SU design-thinking)

**Cochrane Library**

(((human NEXT cent* OR user NEXT cent*) AND (design OR approach)) OR (design NEXT thinking)):ti,ab,kw

**Web of Science**

TS=(human-cent* OR user-cent*) AND TS=(approach OR design)

TS=(“design thinking”)

TS=(health* OR medic* OR clinic*)

**PsycInfo**

(((human-cent* OR user-cent*) AND (design or approach)) OR design thinking).ti,ab,hw,id

(health* OR medic* OR clinic*).ti,ab,hw,id

**Sociological Abstracts**

((human-cent* OR user-cent*) AND (approach OR design))

design-thinking

(health* OR medic* OR clinic*)
