# Supplementary material for: The Application of Human-Centered Design Approaches in Health Research and Innovation: A Narrative Review of Current Practices
Source: JMIR Mhealth Uhealth. 2021 Dec 6;9(12):e28102. doi: 10.2196/28102 (PMC8691403; doi:10.2196/28102)
Supplement: Multimedia Appendix 3 [file mhealth_v9i12e28102_app3.doc]

Supplement 3: Descriptions of the design method included in the review.

| **Method** | **Description** | **Objective** |
| --- | --- | --- |
| Storytelling | A communication tool to share information. | To get insight into user experiences and build empathy. |
| Metaphors | The essence of a metaphor is understanding and experiencing one thing in terms of another. | To generate new ideas, solve problems, and stimulate creativity. |
| Persona’s | Fictional characters of user types or profiles. | To support decision making throughout the design process. |
| Experience Mapping | A visualization of the end-to-end user experience that an average user will go through in order to accomplish a goal. | To understand a general human behavior and create a baseline understanding of an experience. |
| Participatory workshops | An interactive setting which involves multiple stakeholders working actively towards a particular objective. | To create design ideas and acquire understanding of users’ needs and desires. |
| Use case scenario’s | A set of actions, services and functions that a product, system or service needs to perform in a particular context of use. | To describe the actions that a user will perform using the target product, system or service. |
| Decision matrix | A technique to define attributes and weigh them given a relative ranking among design alternatives. | To compare design solutions against one another. To identify the strongest design solution using specific criteria based on project requirements. |
| MoSCoW method | A prioritization technique. | To reach a common understanding with stakeholders on the importance of each design requirement. |
| House of Quality | A product planning matrix. | To show how user requirements relate to the methods used to achieve those requirements. |
| Goal, Question, Metric (GQM) Approach | A goal-oriented measurement for software organization. | To design scenario questions for software process patterns. |
| Role play | A representation technique. | To prototype a service or product idea. |
| User journey mapping | A visualization technique. | To understand a user’s relationship with a product or service of time and across different channels. |
| Intervention mapping | A protocol for developing effective interventions. | To describe the path from problem identification to problem solving. |
| System mapping | A visualization technique. | To visualize all actors and components involved in a service delivery. |
| Low functional prototype | A quick mock-up of ideas using simple assets and materials readily available on spot. | To make ideas tangible and start discussions to iterate on it. |
| High functional prototype | A simulation of the product or service experience by real interaction with one or more components. | To test the product or service, observe the overall user experience and refine design specifications. |
| Use case diagram | A diagram with actors, use cases and their relationships, used to model the system/subsystem of a application. | To visualize functional requirements of a system. |
| Brainstorm | A method to generate ideas and engage stakeholders. | To craft new ideas and creative solutions to a problem. |
| Round Robin | A technique for generating and developing ideas during group brainstorms. | To build off consecutive contributions by each participant. |
| Voting | A technique to democratically prioritize items or make decisions in a group setting. | To democratically support decision making processes. |
| Round table discussions | A deliberative technique to exchange ideas. | To support equal participation of stakeholder on a given topic. |
| Sketching | A visualization technique. | To spur ideas and conversations. |
| Visual mind maps | A visualization technique. | To represent an idea or topic and its subtopics. |
| Storyboarding | A visualization technique. | To visualize the design concept from start to finish. |
| User narratives | A representation of small instances in people’s lives. | To get insight into the use experience and build empathy. |
| Heuristic evaluation | A usability inspection method. | To identify usability problems in the user interface design. |
| Task analysis | A method for breaking a task down into several elements. | To examine the relationship between elements. |
| SWOT | A situation analysis tool. | To derive appropriate strategic solution alternatives to achieve a goal. |
| Wizard of Oz method | A method for prototype testing. | To collect user experiences for further development. |
| Card sorting | A deck of cards with a single word or image. | To identity what is most important to users. |
| Weekly sprints | A time-constrained process to help design teams clearly define goals, validating assumptions and deciding on a design roadmap. | To help teams define clear goals, validate assumptions and decide on a design roadmap. |
| Simulations | An approximate imitation or model of the operation of a process or system for testing, training and optimization. | To test design considerations or specifications with potential users. |
| Cognitive walkthrough | A usability evaluation method. | To understand the users capability to carry out tasks in a given system. |
| Value vs Effort matrix | A prioritization matrix. | to support decision making and identify the value of ideas in relation to the resources needed to realize it. |
