# Supplementary material for: The Application of Human-Centered Design Approaches in Health Research and Innovation: A Narrative Review of Current Practices
Source: JMIR Mhealth Uhealth. 2021 Dec 6;9(12):e28102. doi: 10.2196/28102 (PMC8691403; doi:10.2196/28102)
Supplement: Multimedia Appendix 4 [file mhealth_v9i12e28102_app4.doc]

****Supplement 4:**** The MMAT Checklist

| ****Type of mixed methods study component or primary study**** | ****Methodological quality criteria**** | ****Responses**** | | |
| --- | --- | --- | --- | --- |
|  |  | ****Yes**** | ****No**** | ****Can’t tell**** |
| ****Screening questions**** | - 1. **Are there clear research questions?** |  |  |  |
|  | - 1. **Do the collected data allow address the research questions?** |  |  |  |
| ****Qualitative**** | **1.1 Is the qualitative approach appropriate to answer the research question?** |  |  |  |
|  | **1.2 Is Are the qualitative data collection methods adequate to address the research question?** |  |  |  |
|  | **1.3 Are the findings adequately derived from the data?** |  |  |  |
|  | **1.4 Is the interpretation of results sufficiently substantiated by data?** |  |  |  |
|  | **1.5 Is there coherence between qualitative data sources, collection, analysis and interpretation?** |  |  |  |
| ****Quantitative randomized controlled trials**** | 2.1 Is randomization appropriately performed? |  |  |  |
|  | 2.2 Are the groups comparable at baseline? |  |  |  |
|  | 2.3 Are there complete outcome data? |  |  |  |
|  | 2.4 Are outcome assessors blinded to the intervention provided? |  |  |  |
|  | 2.5 Did the participants adhere to the assigned intervention? |  |  |  |
| ****Quantitative non-randomized**** | 3.1 Are the participants representative of the target population? |  |  |  |
|  | 3.2 Are measurements appropriate regarding both the outcome and intervention (or exposure)? |  |  |  |
|  | 3.3 Are there complete outcome data? |  |  |  |
|  | 3.4 Are the confounders accounted for in the design and analysis? |  |  |  |
|  | 3.5 During the study period, is the intervention administered (or exposure occurred) as intended? |  |  |  |
| ****Quantitative descriptive**** | 4.1 Is the sampling strategy relevant to address the research question? |  |  |  |
|  | 4.2 Is the sample representative of the target population? |  |  |  |
|  | 4.3 Are the measurements appropriate? |  |  |  |
|  | 4.4 Is the risk of nonresponse bias low? |  |  |  |
|  | 4.5 Is the statistical analysis appropriate to answer the research question? |  |  |  |
| ****Mixed methods**** | 5.1 Is there an adequate rationale for using a mixed methods design to address the research question? |  |  |  |
|  | 5.2 Are the different components of the study effectively integrated to answer the research question? |  |  |  |
|  | 5.3 Are the outputs of the integration of qualitative and quantitative components adequately interpreted? |  |  |  |
|  | 5.4 Are divergences and inconsistencies between quantitative and qualitative results adequately addressed? |  |  |  |
|  | 5.5 Do the different components of the study adhere to the quality criteria of each tradition of the methods involved? |  |  |  |

Supplemental Digital Appendix 2. Results MMAT Screening

| Study | | MMAT Criteria items* | | | | | | | | | | | | | | | | |
| --- | --- | --- | --- | --- | --- | --- | --- | --- | --- | --- | --- | --- | --- | --- | --- | --- | --- | --- |
|  |  | Screening | | Qualitative | | | | | Quantitative | | | | | Mixed Method | | | | |
| **Author** | **Year** | **0.1** | **0.2** | **1.1** | **1.2** | **1.3** | **1.4** | **1.5** | **4.1** | **4.2** | **4.3** | **4.4** | **4.5** | **5.1** | **5.2** | **5.3** | **5.4** | **5.5** |
| Bae, J.[25] | 2009 | 3 | 3 | 3 | 3 | 3 | 3 | 3 | 3 | 3 | 3 | 3 | 3 | 3 | 3 | 3 | 3 | 3 |
| Birnie, K. A.[26] | 2019 | 3 | 3 | 1 | 3 | 1 | 1 | 1 | 3 | 3 | 3 | 3 | 3 | 3 | 3 | 3 | 3 | 3 |
| Brox, E.[27] | 2017 | 3 | 3 | 3 | 3 | 3 | 3 | 3 | 3 | 3 | 3 | 3 | 3 | 3 | 3 | 3 | 3 | 3 |
| Cairn, N.[28] | 2017 | 3 | 3 | 3 | 3 | 3 | 3 | 3 | 3 | 3 | 3 | 3 |  | 3 | 3 | 3 | 3 | 3 |
| Carey-Smith, B.E.[29] | 2013 | 3 | 3 | 1 | 3 | 3 | 3 | 3 |  |  |  |  |  |  |  |  |  |  |
| Caro, K.[30] | 2017 | 3 | 3 | 3 | 3 | 3 | 3 | 3 |  |  |  |  |  |  |  |  |  |  |
| Catalani, C.[31] | 2014 | 3 | 3 | 3 | 3 | 3 | 3 | 3 | 3 | 3 | 3 | 3 | 3 | 3 | 3 | 3 | 3 | 3 |
| Cawood, T.[32] | 2015 | 3 | 3 | 3 | 3 | 3 | 3 | 3 |  |  |  |  |  |  |  |  |  |  |
| Civan-Hartlzer, A.[33] | 2010 | 3 | 3 | 1 | 3 | 3 | 3 | 3 |  |  |  |  |  |  |  |  |  |  |
| Connelly, K.[34] | 2016 | 3 | 3 | 3 | 3 | 3 | 3 | 3 | 3 | 3 | 3 | 3 | 3 | 3 |  | 3 | 3 | 3 |
| Crespin, O.M.[35] | 2018 | 3 | 3 | 3 | 3 | 3 | 3 | 3 | 3 | 3 | 3 | 3 | 3 | 3 | 3 | 3 | 3 | 3 |
| Curtis, K.E.[36] | 2015 | 3 | 3 | 1 | 1 | 1 | 1 | 1 | 3 | 3 | 3 | 3 | 3 | 3 | 3 | 3 | 3 | 3 |
| Dabbs de Vito, A.[37] | 2009 | 3 | 3 | 3 | 3 | 3 | 3 | 3 | 3 | 3 | 3 | 3 | 3 | 3 | 3 | 3 | 3 | 3 |
| Das. A.[38] | 2013 | 3 | 3 | 3 | 3 | 3 | 3 | 3 | 3 | 3 | 3 | 3 | 3 | 3 | 3 | 3 | 3 | 3 |
| Davies, A.[39] | 2020 | 3 | 3 | 3 | 3 | 3 | 3 | 3 | 3 | 3 | 3 | 3 | 3 | 3 | 3 | 3 | 3 | 3 |
| Dijkstra. N.[40] | 2018 | 3 | 3 | 3 | 3 | 3 | 3 | 3 | 3 | 3 | 3 | 3 | 3 | 3 | 3 | 3 | 3 | 3 |
| Eberhart, A.[41] | 2019 | 3 | 3 | 3 | 3 | 3 | 3 | 3 |  |  |  |  |  |  |  |  |  |  |
| Erol Barkana, D.[42] | 2013 | 3 | 3 | 3 | 3 | 3 | 3 | 3 |  |  |  |  |  |  |  |  |  |  |
| Erwin, K.[43] | 2019 | 3 | 3 | 3 | 3 | 3 | 3 | 3 |  |  |  |  |  |  |  |  |  |  |
| Ettinger, K.[44] | 2016 | 1 | 3 | 3 | 3 | 3 | 3 | 3 | 3 | 3 | 3 | 3 | 3 | 3 | 3 | 3 | 3 | 3 |
| Fabri, M.[45] | 2016 | 3 | 3 | 3 | 3 | 3 | 3 | 3 | 3 | 3 | 3 | 3 | 3 | 3 | 3 | 3 | 3 | 3 |
| Farinango, C.[46] | 2018 | 3 | 3 | 3 | 3 | 3 | 3 | 3 | 3 | 3 | 3 | 3 | 3 | 3 | 3 | 3 | 3 | 3 |
| Ferris, T.[47] | 2013 | 3 | 3 | 3 | 3 | 3 | 3 | 3 |  |  |  |  |  |  |  |  |  |  |
| Foley, K.[48] | 2019 | 3 | 3 | 3 | 3 | 3 | 3 | 3 |  |  |  |  |  |  |  |  |  |  |
| Fortuna, K.[49] | 2017 | 3 | 3 | 3 | 3 | 3 | 3 | 3 |  |  |  |  |  |  |  |  |  |  |
| Furberg, R.[50] | 2018 | 3 | 3 | 3 | 3 | 3 | 3 | 3 |  |  |  |  |  |  |  |  |  |  |
| Gacnik, M.[51] | 2017 | 3 | 3 | 3 | 3 | 3 | 3 | 3 | 3 | 3 | 3 | 3 | 3 | 3 | 3 | 3 | 3 | 3 |
| Garvelink, M.[52] | 2016 | 3 | 3 | 3 | 3 | 3 | 3 | 3 | 3 | 3 | 3 | 3 | 3 | 3 | 3 | 3 | 3 | 3 |
| Garvelink, M.[53] | 2017 | 3 | 3 | 3 | 3 | 3 | 3 | 3 |  |  |  |  |  |  |  |  |  |  |
| Garvin, J.[54] | 2019 | 3 | 3 | 3 | 3 | 3 | 3 | 3 | 3 | 3 | 3 | 3 | 3 | 3 | 3 | 3 | 3 | 3 |
| Garzo, A.[55] | 2018 | 3 | 3 | 3 | 3 | 3 | 3 | 3 | 3 | 3 | 3 | 3 | 3 | 3 | 3 | 3 | 3 | 3 |
| Gaynor, M.[56] | 2020 | 3 | 3 | 3 | 3 | 3 | 3 | 3 |  |  |  |  |  |  |  |  |  |  |
| Gill, R.K.[57] | 2019 | 3 | 3 | 3 | 3 | 3 | 3 | 3 | 3 | 3 | 3 | 3 | 3 | 3 | 3 | 3 | 3 | 3 |
| Giunti, G.[58] | 2018 | 3 | 3 | 3 | 3 | 3 | 3 | 3 |  |  |  |  |  |  |  |  |  |  |
| Godinho, R.[59] | 2014 | 3 | 3 | 3 | 3 | 3 | 3 | 3 | 3 | 3 | 3 | 3 | 3 | 3 | 3 | 3 | 3 | 3 |
| Gould, C.[60] | 2020 | 3 | 3 | 3 | 3 | 3 | 3 | 3 | 3 | 3 | 3 | 3 | 3 | 3 | 3 | 3 | 3 | 3 |
| Green, R.[61] | 2015 | 3 | 3 | 3 | 3 | 3 | 3 | 3 |  |  |  |  |  |  |  |  |  |  |
| Griffin, L.[62] | 2019 | 3 | 3 | 1 | 3 | 3 | 3 | 3 |  |  |  |  |  |  |  |  |  |  |
| Grossman, L.[63] | 2018 | 1 | 1 | 1 | 3 | 1 | 1 | 1 | 3 | 3 | 3 | 3 | 3 | 3 | 1 | 3 | 3 | 3 |
| Hafiz, P.[64] | 2019 | 3 | 3 | 3 | 3 | 3 | 3 | 3 | 3 | 3 | 3 | 3 | 3 | 3 | 3 | 3 | 3 | 3 |
| Hardy, A.[65] | 2018 | 3 | 3 | 3 | 3 | 3 | 3 | 3 |  |  |  |  |  |  |  |  |  |  |
| Harte, R.[66] | 2017 | 3 | 3 | 3 | 3 | 3 | 3 | 3 | 3 | 3 | 3 | 3 | 3 | 3 | 3 | 3 | 3 | 3 |
| Hartlzer, A.[67] | 2015 | 3 | 3 | 3 | 3 | 3 | 3 | 3 | 3 | 3 | 3 | 3 | 3 | 3 | 3 | 3 | 3 | 3 |
| Herschman, J.[68] | 2014 | 3 | 3 | 3 | 3 | 3 | 3 | 3 |  |  |  |  |  |  |  |  |  |  |
| Horsky, J.[69] | 2016 | 3 | 3 | 3 | 3 | 3 | 3 | 3 |  |  |  |  |  |  |  |  |  |  |
| Huberty, J.[70] | 2016 | 1 | 3 | 1 | 1 | 3 | 3 | 3 | 1 | 1 | 3 | 3 | 3 | 3 | 3 | 3 | 3 | 3 |
| Isenberg, S.[71] | 2018 | 3 | 3 | 3 | 3 | 3 | 3 | 3 | 3 | 3 | 3 | 3 | 3 | 3 | 3 | 3 | 3 | 3 |
| Jonhston, S.[72] | 2017 | 3 | 3 | 3 | 3 | 3 | 3 | 3 | 3 | 3 | 3 | 3 | 3 | 3 | 3 | 3 | 3 | 3 |
| Lan Hing Ting, K.[73] | 2020 | 1 | 1 | 1 | 3 | 3 | 3 | 3 | 3 | 3 | 3 | 3 | 3 | 3 | 3 | 3 | 3 | 3 |
| Luna, D.[74] | 2017 | 3 | 3 | 3 | 3 | 3 | 3 | 3 | 3 | 3 | 3 | 3 | 3 | 3 | 3 | 3 | 3 | 3 |
| Ma, W.[75] | 2007 | 3 | 3 | 3 | 3 | 3 | 3 | 3 |  |  |  |  |  |  |  |  |  |  |
| Madrigal -Cadavid, J.[76] | 2019 | 3 | 3 | 3 | 3 | 3 | 3 | 3 |  |  |  |  |  |  |  |  |  |  |
| Marker, A.[77] | 2019 | 3 | 3 | 3 | 3 | 3 | 3 | 3 | 3 | 3 | 3 | 3 | 3 | 3 | 3 | 3 | 3 | 3 |
| Marko-Holguin, M.[78] | 2019 | 3 | 3 | 3 | 3 | 3 | 3 | 3 | 3 | 3 | 3 | 3 | 3 | 3 | 3 | 3 | 3 | 3 |
| Martin, S.[79] | 2018 | 3 | 3 | 3 | 3 | 3 | 3 | 3 |  |  |  |  |  |  |  |  |  |  |
| McGinn, C.[80] | 2018 | 3 | 3 | 1 | 3 | 3 | 3 | 3 |  |  |  |  |  |  |  |  |  |  |
| McMullen, C.[81] | 2018 | 3 | 3 | 3 | 3 | 3 | 3 | 3 |  |  |  |  |  |  |  |  |  |  |
| Melnick, E.[82] | 2017 | 3 | 3 | 3 | 3 | 3 | 3 | 3 |  |  |  |  |  |  |  |  |  |  |
| Nunez-Nava, J.[83] | 2017 | 3 | 3 | 3 | 3 | 3 | 3 | 3 | 3 | 3 | 3 | 3 | 3 | 3 | 3 | 3 | 3 | 3 |
| Person, B.[84] | 2016 | 3 | 3 | 3 | 3 | 3 | 3 | 3 |  |  |  |  |  |  |  |  |  |  |
| Petersen, M.[85] | 2017 | 3 | 3 | 3 | 3 | 3 | 3 | 3 |  |  |  |  |  |  |  |  |  |  |
| Ragouzeos, D.[86] | 2019 | 3 | 3 | 3 | 3 | 3 | 3 | 3 |  |  |  |  |  |  |  |  |  |  |
| Ray, J.[87] | 2019 | 3 | 3 | 3 | 3 | 3 | 3 | 3 |  |  |  |  |  |  |  |  |  |  |
| Rothgangel, A.[88] | 2017 | 1 | 3 | 1 | 1 | 3 | 3 | 3 | 1 | 1 | 3 | 3 | 3 | 3 | 3 | 3 | 3 | 3 |
| Salmon, M.[89] | 2015 | 3 | 3 | 3 | 3 | 3 | 3 | 3 | 3 | 3 | 3 | 3 | 3 | 3 | 3 | 3 | 3 | 3 |
| Schild, S.[90] | 2019 | 1 | 3 | 1 | 3 | 3 | 3 | 3 | 3 | 3 | 3 | 3 | 3 | 3 | 3 | 3 | 3 | 3 |
| Sedlmayr, B.[91] | 2019 | 3 | 3 | 3 | 3 | 3 | 3 | 3 | 3 | 3 | 3 | 3 | 3 | 3 | 3 | 3 | 3 | 3 |
| Seeber, L.[92] | 2015 | 1 | 3 | 3 | 3 | 3 | 3 | 3 |  |  |  |  |  |  |  |  |  |  |
| Sonney, J.[93] | 2019 | 3 | 3 | 3 | 3 | 3 | 3 | 3 |  |  |  |  |  |  |  |  |  |  |
| Srinivas, P.[94] | 2019 | 3 | 3 | 3 | 3 | 3 | 3 | 3 | 3 | 3 | 3 | 3 | 3 | 3 | 3 | 3 | 3 | 3 |
| Stevens, A.[95] | 2018 | 3 | 3 | 3 | 3 | 3 | 3 | 3 |  |  |  |  |  |  |  |  |  |  |
| Taylor, D.[96] | 2003 | 3 | 3 | 3 | 3 | 3 | 3 | 3 | 3 | 3 | 3 | 3 | 3 | 3 | 3 | 3 | 3 | 3 |
| Timmerman, J.[97] | 2016 | 3 | 3 | 3 | 3 | 3 | 3 | 3 | 3 | 3 | 3 | 3 | 3 | 3 | 3 | 3 | 3 | 3 |
| Tucke Edmonds, B.[98] | 2019 | 3 | 3 | 3 | 3 | 3 | 3 | 3 | 3 | 3 | 3 | 3 | 3 | 3 | 3 | 3 | 3 | 3 |
| van der Weegen, S.[99] | 2013 | 1 | 3 | 3 | 3 | 3 | 3 | 3 |  |  |  |  |  |  |  |  |  |  |
| Vechakul, J.[100] | 2015 | 3 | 3 | 3 | 3 | 3 | 3 | 3 |  |  |  |  |  |  |  |  |  |  |
| Vermeulen, J.[101] | 2013 | 3 | 3 | 3 | 3 | 3 | 3 | 3 | 3 | 3 | 3 | 3 | 3 | 3 | 3 | 3 | 3 | 3 |
| Vilardaga, R.[102] | 2018 | 3 | 3 | 3 | 3 | 3 | 3 | 3 | 3 | 3 | 3 | 3 | 3 | 3 | 3 | 3 | 3 | 3 |
| Wachtler, C.[103] | 2018 | 3 | 3 | 3 | 3 | 3 | 3 | 3 |  |  |  |  |  |  |  |  |  |  |
| Willard, S.[104] | 2018 | 3 | 3 | 3 | 3 | 3 | 3 | 3 | 3 | 3 | 3 | 3 | 3 | 3 | 3 | 3 | 3 | 3 |
| Woodard, T.[105] | 2018 | 3 | 3 | 3 | 3 | 3 | 3 | 3 | 3 | 3 | 3 | 3 | 3 | 3 | 3 | 3 | 3 | 3 |
| Wysocki, T.[106] | 2018 | 3 | 3 | 3 | 3 | 3 | 3 | 3 |  |  |  |  |  |  |  |  |  |  |

*Score: 1 = Yes, 2 = No, 3 = Can’t tell
